# Telerehabilitation to Support Post-Discharge Recovery in Patients with Heart Disease

**DOI:** 10.14789/ejmj.JMJ25-0014-P

**Published:** 2025-07-31

**Authors:** TETSUYA TAKAHASHI, MIHO YOKOYAMA, MASAKAZU SAITOH, KOTARO IWATSU, TOMOYUKI MORISAWA, TOSHIYUKI FUJIWARA, HIROYUKI DAIDA

**Affiliations:** 1 Department of Physical Therapy, Faculty of Health Science, Juntendo University, Tokyo, Japan; 1 Department of Physical Therapy, Faculty of Health Science, Juntendo University, Tokyo, Japan; 2 Department of Cardiovascular Biology and Medicine, Juntendo University Graduate School of Medicine, Tokyo, Japan; 2 Department of Cardiovascular Biology and Medicine, Juntendo University Graduate School of Medicine, Tokyo, Japan; 3 Department of Rehabilitation Medicine, Juntendo University Graduate School of Medicine, Tokyo, Japan; 3 Department of Rehabilitation Medicine, Juntendo University Graduate School of Medicine, Tokyo, Japan

**Keywords:** telerehabilitation, heart disease, digital health, information and communication technologies, tokyo public lecture

## Abstract

In recent years, advances in medical technology have shortened the length of hospital stays for patients with heart disease. However, concerns about recurrence after discharge may lead patients to restrict their activities. Cardiac rehabilitation is particularly important for preventing recurrence and improving quality of life. Until now, outpatient rehabilitation has been the standard approach. However, research is advancing to enable patients to continue rehabilitation safely at home through telerehabilitation. Tele-cardiac rehabilitation involves real-time, two-way communication with physicians and rehabilitation specialists via smartphones or computers. By monitoring electrocardiograms, heart rate, fatigue levels, and other parameters, personalized guidance can be provided to each patient. We believe this system can reduce patients' anxiety and expand their range of activities after discharge. This paper explains the background behind the growing interest in tele-cardiac rehabilitation, the types of tele-cardiac rehabilitation, its benefits, and future challenges.

## Introduction

Cardiovascular disease (CVD) remains the leading cause of death and a primary contributor to healthcare utilization worldwide. As populations age, the prevalence of chronic heart conditions such as heart failure, ischemic heart disease, and arrhythmias is increasing. Acute treatment strategies have improved survival, but these advances have also resulted in a larger population of patients living with chronic cardiac impairment. Following hospitalization, many patients face a significant drop in their physical function, a condition referred to as hospitalization-associated disability (HAD)^1)^. This decline in ability to perform activities of daily living can persist for months and significantly impact quality of life.

While cardiac rehabilitation (CR) programs are effective in mitigating these declines, participation rates remain alarmingly low^2)^. In Japan, as well as globally, outpatient CR programs are often underutilized due to limited access, insufficient referrals, low awareness among patients and providers, and logistical challenges such as transportation difficulties and financial costs^3)^. Furthermore, the psychological impact of a cardiac event can lead to fear-avoidance behavior, where patients are hesitant to engage in physical activity, further compounding the risk of functional decline^4)^.

The COVID-19 pandemic highlighted the vulnerabilities of traditional healthcare delivery models, particularly for patients with chronic conditions. In this context, tele-cardiac rehabilitation (tele-CR) has emerged as a viable and necessary alternative^5)^. By utilizing digital health tools, remote monitoring, and virtual communication, tele-CR extends the reach of rehabilitation programs into the patient's home, promoting continuity of care and individualized support.

## Challenges after discharge in cardiac patients

Hospital discharge is a vulnerable period characterized by a transition from high-intensity inpatient care to low-contact outpatient management. During this time, patients are expected to self-manage complex medication regimens, adhere to lifestyle modifications, and initiate physical recovery -all without the continuous supervision they experienced in the hospital. For elderly patients, those with cognitive impairment, and those with limited social support, this can result in rapid deconditioning, medication non-adherence, and avoidable readmissions^6)^.

In Japan, the J-PROOF HF study has demonstrated that approximately 37% of older patients hospitalized for heart failure experience HAD at discharge^7)^. HAD is independently associated with increased risk of institutionalization and mortality^8)^. This makes early and sustained rehabilitation a clinical imperative. However, data show that only a minority of patients participate in CR after discharge, and even fewer complete a full course of outpatient sessions^2, 3)^.

Patients encounter multiple barriers to participation: logistical (distance from hospital, transportation issues), financial (cost of attendance), psychological (fear of exertion, lack of motivation), and informational (lack of understanding about the benefits of CR) (Table 1)^9)^. These challenges necessitate innovative models of care that reduce patient burden while ensuring clinical efficacy^5)^.

**Table 1:** Barriers to participation in outpatient cardiac rehabilitation

Category	Barrier
Structural barriers	Residence far from facility
	Competing responsibilities (work, household chores)
	High transportation costs
	High program cost
	Lack of awareness of cardiac rehabilitation
	Lack of family support
	Physician-imposed exercise restriction
Psychological barriers	Dislike of exercise
	Fear of exercise
	Belief that exercise may worsen condition
	Lack of future motivation to exercise
	Belief in ability to self-manage without formal program
	Lack of understanding about the benefits of CR

## Models and technologies in tele-cardiac rehabilitation

Tele-CR is not a monolithic approach but rather a spectrum of care modalities, all designed to replicate the core components of traditional CR─namely exercise training, risk factor management, education, and psychosocial support—within a virtual framework. These models can be broadly classified as follows^5)^ (Table 2).

In the asynchronous model, a self-managed format, patients can complete prescribed exercise sessions on their own schedule, using monitoring devices that transmit biometric data to clinicians for periodic review. This model is resource-efficient and scalable but requires patients to be motivated and technologically capable.

In the synchronous model, patients conduct exercise sessions in real-time with clinicians and healthcare providers via video conferencing platforms. This allows for direct supervision, immediate feedback, and real-time adjustments. It is particularly beneficial for higher-risk patients or those who are new to exercise.

The hybrid model combines the benefits of synchronous and asynchronous models. For example, a patient may attend a live virtual session once a week and complete additional independent exercises throughout the week. Hybrid models offer flexibility and ensure ongoing engagement.

Technologies supporting tele-CR include:

• Wearable ECG and heart rate monitors

• Pulse oximeters and smart blood pressure monitors

• Tablet- or smartphone-based apps for video conferencing

• IoT-connected ergometers and home gym equipment

• Cloud-based platforms for data aggregation and analytics

**Figure 1:**
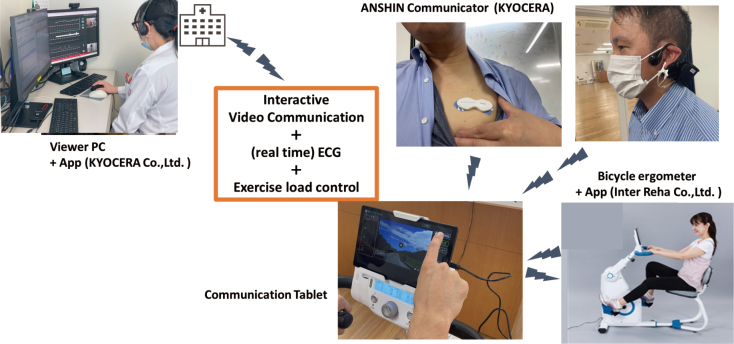
Bicycle ergometer IoT model

Juntendo University has developed customized tele-CR programs utilizing devices such as Heart Monitor, interactive apps, and real-time video assessment. The “bicycle ergometer IoT model” is one such example, integrating biometric monitoring with visual communication for supervised aerobic training (Figure 1).

**Table 2:** Models of tele-cardiac rehabilitation

Model	Description	Patient engagement	Monitoring type
Asynchronous	Self-paced exercise with device-based monitoring	Independent	Delayed (data uploaded)
Synchronous	Real-time video-supervised sessions	Interactive	Real-time
Hybrid	Mix of real-time and self-paced components	Semi-supervised	Mixed

## Clinical evidence and institutional examples

Several studies have established the clinical efficacy of tele-CR. Randomized controlled trials have shown that tele-CR is non-inferior to traditional CR in terms of improving exercise capacity, reducing cardiovascular risk factors, and enhancing health- related quality of life^10, 11)^. In some cases, adherence and completion rates are higher in tele-CR cohorts due to the convenience and personalization of the approach^12)^.

In our previous study, we investigated the safety and efficacy of remote cardiac rehabilitation (Remote- CR) using tablet devices in elderly patients with heart disease^13)^. In this study, 11 patients aged 65 years or older with heart disease were enrolled and compared between a tele-CR group and a conventional center-based rehabilitation (CB-CR) group. Patients in the tele-CR group received weekly real- time video calls for guidance and had their vital signs, such as heart rate and blood pressure, remotely monitored using tablet devices. After a 4-week intervention, patients in the tele-CR group showed improvements in functional indicators such as grip strength, usual walking speed, Short Physical Performance Battery (SPPB), and Basic Checklist (KCL) comparable to those in the CB-CR group. Additionally, tele-CR demonstrated high patient satisfaction and suggested potential for contributing to the maintenance of healthy lifestyle habits. This study demonstrates that remote cardiac rehabilitation is a safe and feasible intervention for older patients.

Furthermore, our other study evaluated the safety and feasibility of tele-cardiac rehabilitation using a remote bio-signal monitoring system. In this study, a 3-month tele-CR was conducted on 9 patients with cardiovascular disease, and significant improvements in exercise tolerance were confirmed. Additionally, no serious issues occurred during remote monitoring, and patients were able to operate the remote system appropriately^14)^.

These findings are echoed by global meta-analyses that show tele-CR to be effective across diverse patient populations. High patient satisfaction, improved adherence, and reduced travel burden make it a compelling option for healthcare systems looking to improve value-based care delivery.

## Benefits and limitations of tele-CR benefits

The benefits and limitations of tele-CR are summarized in the table below (Table 3).

Addressing these barriers requires coordinated efforts including caregiver training, development of intuitive user interfaces, and robust technical support systems. Policymakers must recognize the long-term cost savings and improved outcomes associated with tele-CR when designing reimbursement frameworks.

**Table 3:** Benefits and limitations of tele-cardiac rehabilitation

Category	Description
Benefits	
Accessibility	Expands access to care in rural or underserved areas
Convenience	Reduces travel burden and integrates rehabilitation into daily routines
Continuity of care	Supports long-term engagement and monitoring outside of traditional clinical settings
Scalability	Enables expansion of services without proportional increase in staffing
Personalization	Real-time data allows for individualized, adaptive intervention planning
Limitations	
Digital literacy	Older adults or low-tech users may face difficulty operating digital platforms
Connectivity	Requires stable internet and reliable digital infrastructure
Regulatory issues	Some remote monitoring devices lack formal approval in certain healthcare systems
Reimbursement	Limited insurance coverage or unclear policies restrict widespread adoption
Safety concerns	Delayed detection of emergencies may occur without clear remote monitoring protocols

## Future perspectives in digital health integration

The future of tele-CR lies in its integration with broader digital health ecosystems. Artificial intelligence can be employed to analyze biometric trends, predict exacerbations, and recommend personalized adjustments to rehabilitation protocols. Machine learning algorithms could stratify patients by risk and suggest optimal frequency, intensity, and type of exercise^15)^.

Tele-CR platforms can also be connected to electronic health records, enabling seamless communication among multidisciplinary teams. Integration with tele-nursing, virtual pharmacy consults, dietary counseling, and behavioral therapy will offer patients a comprehensive and cohesive care experience^5)^.

Innovations such as gamified exercise modules, community engagement tools, and wearable haptic feedback devices could further enhance motivation and adherence. Importantly, culturally sensitive and linguistically appropriate content must be developed to ensure inclusivity across patient populations.

Clinical research should continue to evaluate long-term outcomes of tele-CR, including effects on mortality, hospitalization rates, healthcare costs, and patient-reported outcomes^16)^. Health systems should prioritize implementation science to guide best practices for deploying tele-CR at scale.

## Conclusion

Tele-cardiac rehabilitation is a transformative model of care that addresses critical gaps in post- discharge recovery for cardiovascular patients. Its value is especially pronounced in the context of aging societies, workforce shortages, and healthcare system constraints. By removing traditional barriers and enabling tailored interventions in patients' homes, tele-CR can improve adherence, reduce rehospitalization, and support sustainable chronic disease management.

For tele-CR to become a mainstay in cardiac care, concerted action is needed from clinicians, health administrators, technology developers, and policymakers. Standardized protocols, regulatory clarity, equitable access to devices, and integrated care pathways will ensure its successful adoption. As digital health continues to evolve, tele-CR stands at the forefront of a more connected, responsive, and patient-centered healthcare future.

## Author contributions

TT, MY, MS, IK, and TM explored hospitalization-associated disabilities and post-discharge challenges in patients with heart disease and were involved in the development of a model and technology for tele-cardiac rehabilitation. TF provided advice on the development of telerehabilitation technology, and HD oversaw tele-cardiac rehabilitation research and discussed future prospects for digital health research.

## Conflicts of interest statement

This article includes findings derived from collaborative research with Kyocera Corporation and Inter Reha Co., Ltd. The authors declare no other conflicts of interest related to the content of this manuscript.
